# Toll-Like Receptor 8 Agonist and Bacteria Trigger Potent Activation of Innate Immune Cells in Human Liver

**DOI:** 10.1371/journal.ppat.1004210

**Published:** 2014-06-26

**Authors:** Juandy Jo, Anthony T. Tan, James E. Ussher, Elena Sandalova, Xin-Zi Tang, Alfonso Tan-Garcia, Natalie To, Michelle Hong, Adeline Chia, Upkar S. Gill, Patrick T. Kennedy, Kai Chah Tan, Kang Hoe Lee, Gennaro De Libero, Adam J. Gehring, Christian B. Willberg, Paul Klenerman, Antonio Bertoletti

**Affiliations:** 1 Viral Hepatitis Laboratory, Singapore Institute for Clinical Sciences, Agency of Science Technology and Research (A*STAR), Singapore; 2 Program Emerging Infectious Diseases, Duke-NUS Graduate Medical School, Singapore; 3 NIHR Biomedical Research Centre, John Radcliffe Hospital & Peter Medawar Building for Pathogen Research, University of Oxford, Oxford, United Kingdom; 4 Institute of Cell and Molecular Science, Barts and the London School of Medicine & Dentistry, London, United Kingdom; 5 Asian American Liver Centre, Singapore; 6 Experimental Immunology, Department of Biomedicine, University Hospital Basel, Basel, Switzerland; 7 School of Immunity and Infection, College of Medical and Dental Science, University of Birmingham, Edgbaston Birmingham, United Kingdom; Nationwide Children's Hospital, United States of America

## Abstract

The ability of innate immune cells to sense and respond to impending danger varies by anatomical location. The liver is considered tolerogenic but is still capable of mounting a successful immune response to clear various infections. To understand whether hepatic immune cells tune their response to different infectious challenges, we probed mononuclear cells purified from human healthy and diseased livers with distinct pathogen-associated molecules. We discovered that only the TLR8 agonist ssRNA40 selectively activated liver-resident innate immune cells to produce substantial quantities of IFN-γ. We identified CD161^Bright^ mucosal-associated invariant T (MAIT) and CD56^Bright^ NK cells as the responding liver-resident innate immune cells. Their activation was not directly induced by the TLR8 agonist but was dependent on IL-12 and IL-18 production by ssRNA40-activated intrahepatic monocytes. Importantly, the ssRNA40-induced cytokine-dependent activation of MAIT cells mirrored responses induced by bacteria, i.e., generating a selective production of high levels of IFN-γ, without the concomitant production of TNF-α or IL-17A. The intrahepatic IFN-γ production could be detected not only in healthy livers, but also in HBV- or HCV-infected livers. In conclusion, the human liver harbors a network of immune cells able to modulate their immunological responses to different pathogen-associated molecules. Their ability to generate a strong production of IFN-γ upon stimulation with TLR8 agonist opens new therapeutic opportunities for the treatment of diverse liver pathologies.

## Author Summary

The ability of human pathogens, like HBV, HCV or *Plasmodium* spp. to infect the liver might be influenced by its tolerogenic features. However, hepatic tolerance is not absolute since protective immunity can be triggered. Our goal was to define how to deliberately elicit an intrahepatic protective immune response. To achieve this, we purified immune cells residing in the vascular bed of human livers and we probed their reactivity against different pathogen-associated molecules, mimicking signature components of viruses or bacteria. We found that robust production of anti-viral cytokine IFN-γ was induced only by the TLR8 agonist ssRNA40. Mechanistically, ssRNA40 triggered hepatic monocytes to produce IL-12 and IL-18 cytokines, which stimulated IFN-γ production by liver-resident CD161^Bright^ MAIT and CD56^Bright^ NK cells. We also demonstrated that ssRNA40-mediated activation could occur in pathologic (HBV- or HCV-chronically infected) livers and that a similar cytokine-mediated activation of intrahepatic cells could also be triggered upon bacterial infection. Thus, we showed that the liver immune cells can respond vigorously to specific pathogen-associated molecules. The selective production of IFN-γ by liver-resident cells could have therapeutic implications for the treatment of chronic liver infections.

## Introduction

The liver is an essential organ at the center of carbohydrate, lipid and protein metabolisms. It is crucial for clearing toxins and pathogens that reach the circulatory compartment from the gut. The liver is also home to abundant populations of innate immune cells (monocytes, NK and NKT cells) whose local activation needs to be tuned in order to avoid severe liver damage with life-threatening consequences [1], [2]. For these reasons, the immunological environment of the liver has been primarily associated with tolerogenic features: abundance of immunosuppressive cytokines/ligands (e.g., IL-10 or PD-L1), tolerance to LPS stimulation and production of inhibitory enzymes (e.g., arginase) that can suppress immune responses [3], [4]. The ability of pathogens like HBV, HCV and *Plasmodium* spp. to establish persistent infections in the liver can be facilitated by such immunotolerant features.

The hypo-responsiveness of liver-resident immune cells is, however, not absolute and selective triggers are known to activate hepatic NK or CD56^+^ T cells: for example, liver-resident iNKT cells are activated in mice infected with *Borrelia burgdorferi* [5]. Using human immune cells purified from a donor liver for transplant, it was also shown that while Toll-like receptor 4/TLR4 and TLR2 agonists triggered a tolerogenic response and preferential IL-10 production, a TLR3 agonist activated hepatic NK cells through IL-18-mediated stimulation [6].

The difficulty in obtaining a substantial number of intrahepatic human cells has precluded a comprehensive analysis of the signals necessary to activate liver immunity. Furthermore, there is a general problem of translating murine findings to the human liver. The vast population of liver-resident T cells expressing NK markers are mostly composed by MR1-restricted mucosal-associated invariant T (MAIT) cells in humans and not by the classical CD1d-restricted NKT cells, which are abundant in mice [7]. Thus, studies on human hepatic immune cells are crucial. Utilizing intrasinusoidal samples obtained during the procedure preceding living-donor liver transplantation, we characterized the requirements for selective activation of human intrahepatic immune cells and the cellular subsets responsible for inducing a potent immune response in the human liver microenvironment.

## Results

### Selective stimulation of liver intrasinusoidal cells by a TLR8 agonist

**Figure 1:**
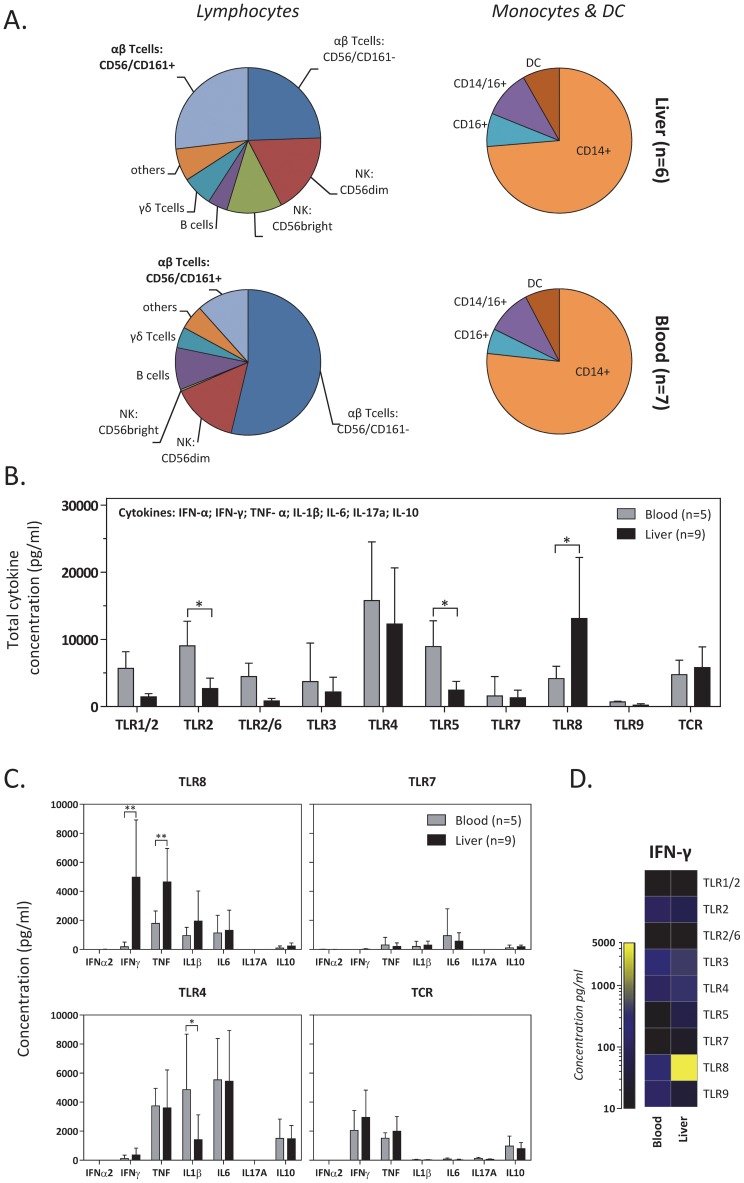
Selective stimulation of liver-derived cells by a TLR8 agonist. *(A)* Pie charts depict the average proportion of different subsets of lymphocytes, monocytes and dendritic cells found in the liver (n = 6) and in the peripheral blood (n = 7) of healthy donors. *(B)* Mean±SD total concentration of cytokines (IFN-α, IFN-γ, TNF-α, IL-1β, IL-6, IL-17a and IL-10) in the supernatant after stimulation of purified lymphocytes isolated from the peripheral blood (n = 5) and liver (n = 9) with the indicated TLR agonist and anti-CD3/CD28-coupled beads. Unstimulated lymphocytes were used to determine the background levels and the background subtracted values are displayed. *(C)* Background subtracted Mean±SD concentrations of individual cytokines quantified in the supernatant of purified lymphocytes isolated from the peripheral blood (n = 5) or liver (n = 9) and stimulated with either TLR8, TLR7 or TLR4 agonist or anti-CD3/CD28-coupled beads. *(D)* Heatmap shows the background subtracted mean concentrations of IFN-γ in the supernatants of blood (n = 5) or liver- derived lymphocytes (n = 9) stimulated with the indicated TLR agonist. * and ** indicates P<0.05 and P<0.01 respectively.

To test whether liver intrasinusoidal cells can be differentially activated by various pathogen-associated molecules, mononuclear cells purified from healthy liver grafts preceding living-donor liver transplantations (called liver-derived cells/LDCs) were stimulated with TLR agonist 1/2, 2, 2/6, 3, 4, 5, 7, 8, or 9 (respectively Pam3CSK4, HKLM, FSL-1, poly(I:C), LPS, flagellin, imiquimod, ssRNA40, or CpG ODN2216) or anti-CD3/CD28-coupled beads (TCR) as a control. We used TLR agonists at the concentration that triggered maximal activation in PBMCs (not shown). After 18 hours of incubation, supernatants were collected and the concentrations of antiviral (IFN-α, IFN- γ), pro-inflammatory (IL-1β, IL-6, IL-17A, TNF-α) and immunosuppressive (IL-10) cytokines were measured. The purity and cell composition of LDCs were recently described in detail [8]. For clarity, the differential composition of lymphocytes and of monocytes and dendritic cells/DCs obtained from the liver (n = 6) or peripheral blood (n = 7) of age-matched healthy subjects are shown again in Fig. 1A. Liver-derived lymphocytes are enriched in CD56^Bright^ NK and T cells expressing NK markers CD56 and CD161 which are mainly mucosal-associated invariant T (MAIT) cells [8]. The frequency of different monocyte subsets (CD14^++^CD16^−^, CD14^+^CD16^+^ and CD14^dim^CD16^+^) and of DCs were in contrast similar in LDCs and PBMCs.

Fig. 1B shows the total production of IFN-α, IFN-γ, IL1β, IL-6, IL-10, TNF-α obtained in PBMCs of 5 healthy subjects and LDCs from 9 healthy liver donors (matched for age). The tested TLR agonists activated higher production of cytokines in PBMCs than LDCs with the single notable exception of the TLR8 agonist ssRNA40. Analysis of the single cytokines produced in ssRNA40-activated LDCs showed a very high quantity of IFN-γ, followed by TNF-α and IL-1β (Fig. 1C). IFN-γ quantity produced by ssRNA40-activated LDCs (∼5000 pg/mL) was higher than the IFN-γ triggered by anti-CD3/CD28-coupled beads (∼3000 pg/mL) and by the other TLR agonists (<500 pg/mL) (Fig. 1C and 1D). ssRNA40-activated LDCs also produced high quantities of IL-1β and TNF-α, but the differences between LDCs and PBMCs were not as dramatic as that observed for IFN-γ: on average 27 times higher in LDCs than PBMCs (Fig. 1C and 1D). The TLR4 agonist LPS elicited also a high production of cytokines in LDCs (Fig. 1B). The pro-inflammatory IL-1β, IL-6 and TNF-α and the immunoregulatory IL-10 cytokines were the most highly produced with levels similar between PBMCs and LDCs (IL-6, TNF-α, IL-10) or higher in PBMCs than LDCs (IL-1β) (Fig. 1C). IFN-α was detectable only at low concentrations (∼63 pg/mL) upon TLR9 activation with production higher in PBMCs than in LDCs (not shown). TLR agonists did not induce production of IL-17A, which was only detectable at low levels in LDCs and PBMCs (∼57 and ∼124 pg/mL, respectively) upon TCR stimulation (not shown).

### MAIT and CD56^Bright^ NK cells are the main IFN-γ-producing sources within ssRNA40-activated LDCs

**Figure 2:**
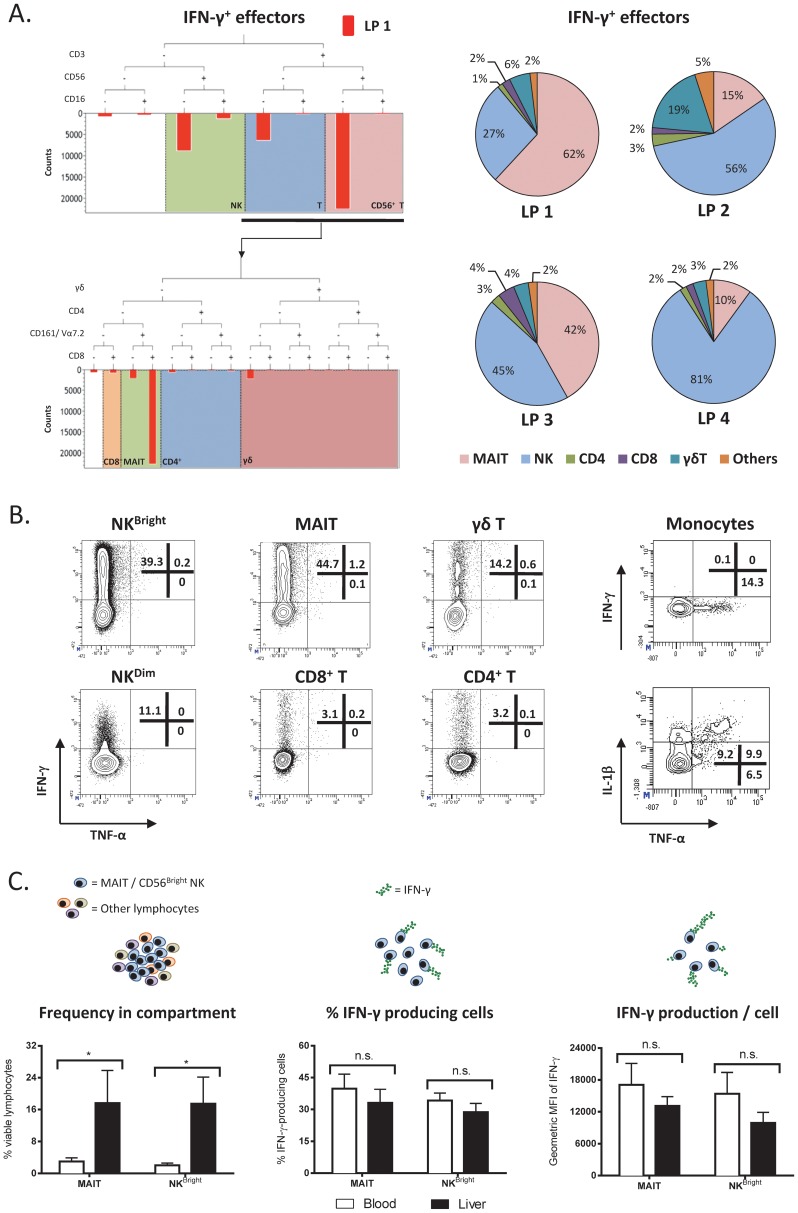
Characterization of intrahepatic immune cell subsets that produce IFN-γ upon TLR8 agonist stimulation. *(A)* The left panel shows a tree plot depicting lymphocyte subsets within the healthy liver compartment that produce IFN-γ upon ssRNA40 stimulation. A representative donor, LP1, is presented. Pie charts illustrate the same data from 4 healthy donors, LP1-LP4. *(B)* Production of IFN-γ, TNF-α and IL-1β by seven immune cell subsets within the liver compartment upon ssRNA40 stimulation. The frequency of cytokine-producing cells within each subset is shown in the quadrants. Representative contour plots of one healthy donor are presented. *(C)* Analysis of IFN-γ production by MAIT and NK^Bright^ cells within the peripheral blood (n = 3) and liver (n = 4) compartments of healthy donors. The left panel illustrates the frequency of MAIT or NK^Bright^ cells represented as a proportion of total viable lymphocytes in each compartment. The middle panel shows the frequency of IFN-γ-producing MAIT or NK^Bright^ cells in each compartment. The right panel shows the production of IFN-γ per cell within the MAIT or NK^Bright^ populations in each compartment. Values are displayed as mean±SD. * and n.s. indicate P<0.05 and P>0.05, respectively.

We next characterized the cellular component responsible for the high IFN-γ, TNF-α and IL-1β production in LDCs after ssRNA40 stimulation. Visualization of cytokine-producing cells was performed by intracellular staining, adding the protein transport inhibitor brefeldin A either immediately or only in the last 5 hours of the stimulation. IFN-γ-producing cells within both the CD3^+^ and CD3^−^ lymphocyte populations were visualized only with the addition of brefeldin A in the final 5 hours as the overnight presence of brefeldin A resulted in a diminished response (not shown). Characterization of the cellular composition of IFN-γ producers based on NK- and T-cell subsets (see tree diagram in Fig. 2A) revealed that despite different frequencies in different individual samples (pie charts in Fig. 2A), three lymphocyte populations were responsible for the majority of IFN-γ production upon ssRNA40 stimulation: CD3^−^CD56^+^CD16^−^ (CD56^Bright^ NK or NK^Bright^ cells), CD3^+^γδ^−^CD4^−^CD161^+^Valpha 7.2^+^ (MAIT cells) and to a lesser extent, CD3^+^γδ^+^ cells (γδ T cells). CD56^Dim^ NK (NK^Dim^ cells) and conventional T cells were, in contrast, weakly activated (Fig. 2A and 2B). Importantly, as shown in the dot plots of a representative sample in Fig. 2B, ssRNA40-activated lymphocytes produced only IFN-γ, while TNF-α and IL-1β were produced by activated monocytes (Fig. 2B).

The higher production of IFN-γ by ssRNA40-activated LDCs in comparison to PBMCs can be explained by the preferential liver compartmentalization of MAIT and NK^Bright^ cells (Fig. 2C first panel). However we also tested whether liver-derived MAIT and NK^Bright^ cells were more efficiently activated than blood-derived cells. As shown in Fig. 2C, the frequency of IFN-γ-producing MAIT or NK^Bright^ subset within the total population present in liver or blood (Fig. 2C second panel) and their capacity to produce IFN-γ, measured by geometric MFI (Fig. 2C third panel) were not different in relation to their anatomical origin. Therefore, the higher production of IFN-γ in the supernatant of ssRNA40-stimulated LDCs in comparison to PBMCs is likely a consequence of the specific enrichment of MAIT and NK^Bright^ cells in the hepatic environment.

### Characterization of ssRNA40-mediated activation of intrahepatic immune cells

We conducted a series of experiments to characterize the mechanism of ssRNA40-mediated activation of intrahepatic immune cells. We first blocked the generation of endolysosomes that are necessary for TLR8 signaling upon ssRNA40 stimulation [9] by using the endosomal acidification inhibitor chloroquine and the vacuolar-type H+ ATPase inhibitor bafilomycin A1. The experiments demonstrated that inhibition of endolysosome acidification indeed blocked ssRNA40-mediated activation (Fig. S1), confirming the essential role of TLR8 in ssRNA40 stimulation. We also confirmed that the production of IFN-γ was dependent on uracil-rich ssRNA40, but not seen with its control, adenine-rich ssRNA41 [10], or its vehicle, Lyovec (Fig. S2).

**Figure 3:**
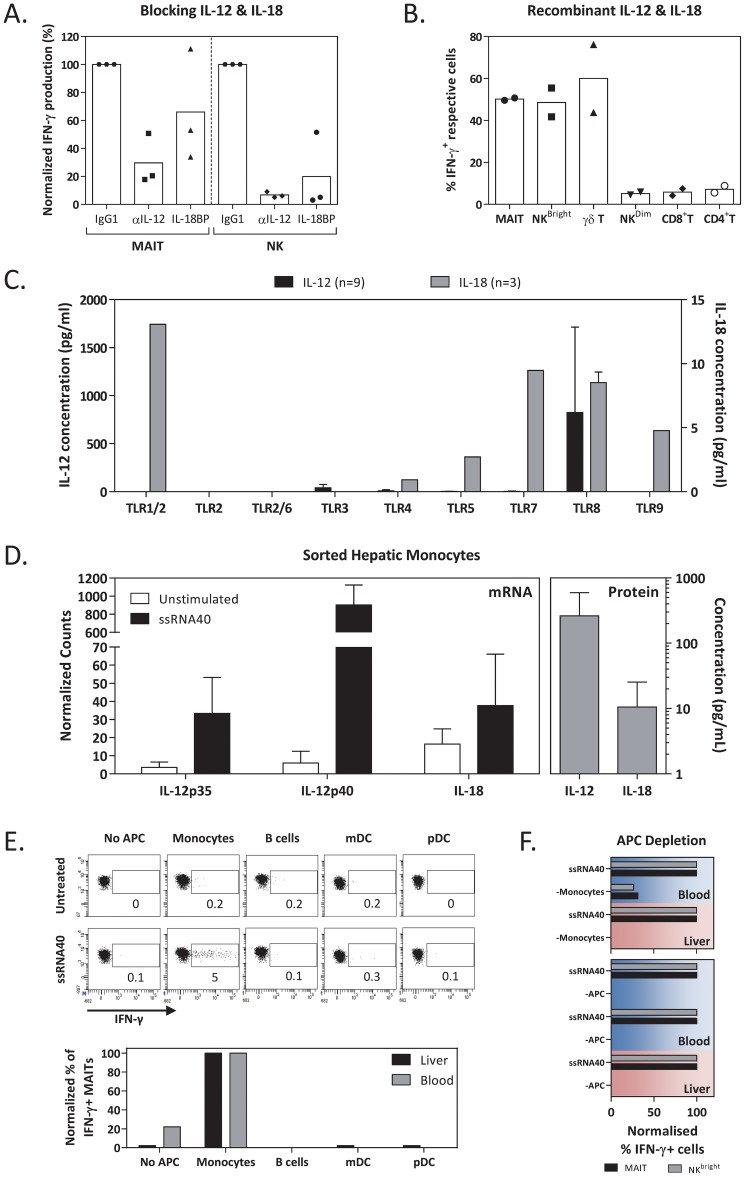
TLR8 agonist activation of liver-derived cells were mediated by IL-12 and IL-18 production from monocytes. *(A)* Frequency of IFN-γ-producing MAIT and NK^Bright^ cells were quantified after stimulation of liver-derived lymphocytes (n = 3) with TLR8 agonist in the absence or presence of IL-12 or IL-18-neutralizing antibodies/proteins. The IFN-γ response was normalized to the isotype control. Bars show the mean response while the individual values were indicated by the symbols. *(B)* Frequency of IFN-γ producing cells within each indicated subset were quantified after stimulation of liver derived lymphocytes (n = 2) with recombinant human IL-12 and IL-18. *(C)* Mean±SD concentrations of IL-12p70 (n = 9) and IL-18 (n = 3) in the supernatants of liver derived lymphocytes after stimulation with the indicated TLR agonists. IL12p70 was significantly increased after ssRNA40 stimulation (p<0.0001). *(D)* Quantification of *IL-12p35*, *IL-12p40* and *IL-18* mRNA levels in sorted hepatic monocytes (n = 2) before and after stimulation with ssRNA40. The corresponding background subtracted supernatant concentrations of IL-12 and IL-18 were also quantified and displayed as grey bars. Error bars show the standard deviation of our data set. *(E)* MAIT cells from the liver or blood were sorted and stimulated with ssRNA40 in the presence or absence of a single subset of autologous and sorted APC (monocytes, B cells, mDC or pDC). Dot plots show the frequency of IFN-γ-producing liver-derived MAIT cells before and after stimulation with ssRNA40 in the presence of the indicated APC subset. The bar chart compares the APC requirement of ssRNA40 stimulated blood- and liver-derived MAIT cells. The frequency of IFN-γ-producing MAIT cells is normalized to that in the presence of monocytes. *(F)* Blood- or liver-derived lymphocytes were stimulated with ssRNA40 after depletion of either APCs (HLA-DR positive) or monocytes. Background subtracted frequency of IFN-γ-producing MAIT and NK^Bright^ cells from each donor are shown as bars. Values are normalized to that in the absence of depletion.

We next analyzed how ssRNA40 activates MAIT and NK^Bright^ cells. A previous study of hepatic NK-cell activation demonstrated that IL-18 and IL-12p70 (hereby stated as IL-12) production by hepatic monocytes is necessary for their activation [6]. MAIT cells are instead known to be principally triggered by microbial riboflavin metabolites presented by MR1 molecules on APCs [11], even though recent studies by others [12] and us [13] indicated that upon overnight co-culture with bacteria, circulating MAIT-cell activation was dependent on the MR1 recognition as well as on the presence of IL-12 and IL-18. We therefore analyzed the role played by IL-12 and IL-18 in the ssRNA40-mediated activation of intrahepatic lymphocytes. LDCs from three different donors were stimulated with ssRNA40 in the presence or absence of anti-IL-12 or anti-IL-18 neutralizing antibodies. Anti-IL-12 or anti-IL-18 antibodies inhibited activation of hepatic MAIT and NK cells (Fig. 3A). Furthermore, addition of recombinant IL-12 and IL-18 to LDCs induced IFN-γ production selectively in MAIT, NK^Bright^ and also γδ T cells, similarly to what observed in ssRNA40-mediated stimulation of LDCs (Fig. 3B).

The importance of IL-12 and IL-18 cytokines in the ssRNA40-mediated activation of intrahepatic immunity was further supported by the observation that across all tested TLR agonists, only ssRNA40 elicited a production of both IL-12 and IL-18 cytokines in LDCs (Fig. 3C). More importantly, ssRNA40 was the only TLR agonist that stimulated a significantly large (mean: 824.2 pg/mL) production of IL-12 (P<0.0001).

Human monocytes, particularly the CD14^dim^CD16^+^ population, have been shown to sense nucleic acids through TLR8 receptors [14]. We therefore directly tested whether hepatic monocytes produce IL-12 and IL-18 upon ssRNA40 stimulation. Hepatic monocytes were sorted from 2 different donors of LDCs and then stimulated with ssRNA40 for 18 hours. IL-12 and IL-18 production were detected both at mRNA and protein levels (Fig. 3D).

To directly test the ability of ssRNA40-activated monocytes to stimulate IFN-γ production by NK and MAIT cells, we sorted different populations of APC and lymphocytes (NK and MAIT) from one normal liver and one healthy peripheral blood. Regardless of their anatomical origin (Fig. 3E), only monocytes were able to stimulate IFN-γ production in NK and MAIT cells.

The ability of intrahepatic and circulating monocytes to activate NK and MAIT cells after ssRNA40 activation was further confirmed in depletion experiments. These were performed in cells purified in two distinct healthy liver and blood samples. Depletion of monocytes or total APC from bulk cell populations abolished the ssRNA40-mediated activation of MAIT and NK cells (Fig. 3F).

### Activation of hepatic MAIT cells by IL-12 and IL-18 upon bacterial infection

**Figure 4:**
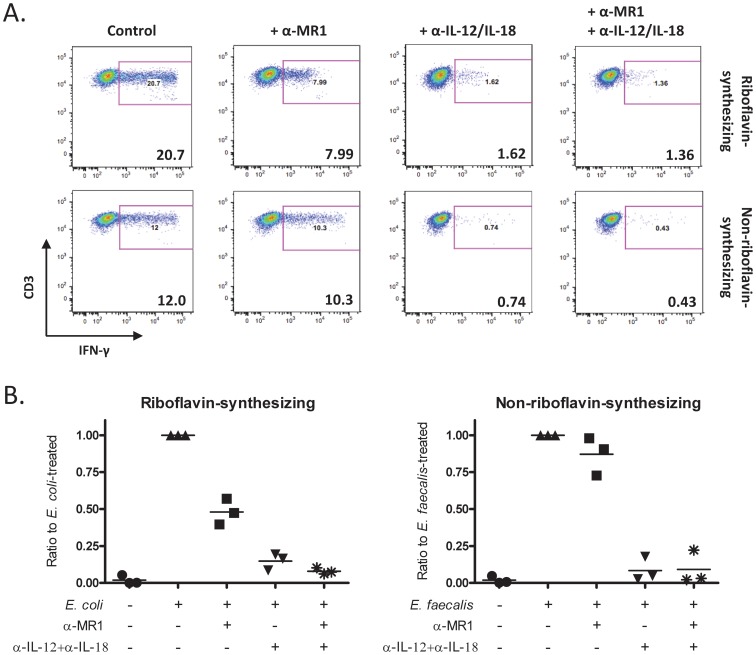
Activation of MAIT cells upon overnight bacterial infection. *(A)* Representative dot plots from one healthy donor of IFN-γ production by liver-derived MAIT cells upon co-culture for 20 hours with bacteria which synthesize riboflavin (*Escherichia coli*) or bacteria which do not synthesize riboflavin (*Enterococcus faecalis*) in the presence or absence of blocking antibodies against MR1 and/or IL-12 and IL-18. *(B)* The frequency of IFN-γ-producing MAIT cells when co-cultured with either riboflavin-synthesizing or non-riboflavin-synthesizing bacteria in the presence of blocking antibody is given relative to that in the absence of any blocking antibody, the latter of which is set as 1. Three healthy donors were tested with each symbol representing one donor.

We recently demonstrated that overnight bacterial stimulation of healthy PBMCs activated MAIT cells to produce IFN-γ directly via MR1 as well as indirectly via IL-12- and IL-18-dependent mechanisms [13]. To test if this finding was true in LDCs, we stimulated bulk population with riboflavin- and non-riboflavin-synthesizing bacteria (*E. coli* and *E. faecalis* respectively): only riboflavin-synthesizing bacteria can produce a ligand presented by MR1 [11]. The bacterial stimulation was performed for 20 hours in the presence or absence of blocking antibodies against MR1 or IL-12 and IL-18. Importantly, we observed that upon overnight co-culture with riboflavin-synthesizing bacteria, hepatic MAIT cells were activated by both IL-12 and IL-18 cytokines and by MR1-restricted ligand (Fig. 4A and 4B). In contrast, activation by non-riboflavin-synthesizing bacteria was entirely dependent upon IL-12 and IL-18. Similar results were obtained using THP1 cells, a monocytic cell line, as APCs. Consistent with our findings with blood-derived MAIT cells [13], early activation (5 hours) of liver-derived MAIT cells with riboflavin-synthesizing bacteria was MR1-dependent, while later activation (20 hours) was dependent upon both MR1 and IL-12 and IL-18 (Fig. S3). Similarly, experiments using non-riboflavin-synthesizing bacteria reinforced the important role of cytokines in MAIT-cell activation to produce IFN-γ.

### Activation of hepatic MAIT cells by ssRNA40 mimics MAIT-cell activation upon bacterial infection

**Figure 5:**
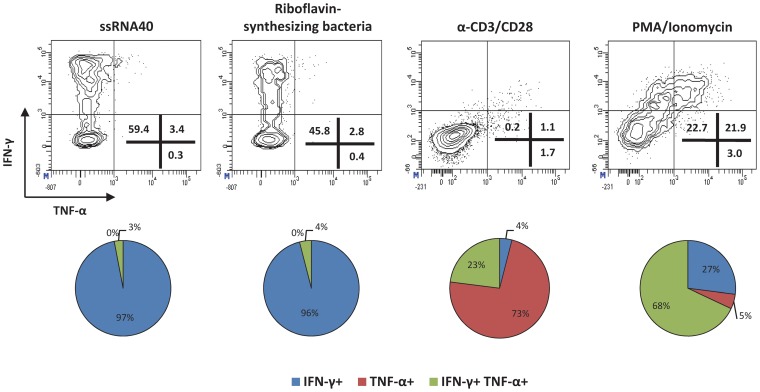
TLR8 stimulation mimics MAIT cell activation upon bacterial infection. (*Upper panels*) Representative contour plots illustrate the production of IFN-γ and TNF-α by MAIT cells upon overnight stimulation of liver-derived cells with ssRNA40 (5 µg/mL), riboflavin-synthesizing bacteria (*Pseudomonas aeruginosa*), anti-CD3/CD28-coupled beads or PMA/Ionomycin. (*Lower panels*) Pie charts depicting the proportion of IFN-γ or TNF-α monoproducers and IFN-γ/TNF-α double producers among activated MAIT cells are shown. Data expressed as means obtained from two healthy donors.

To further analyze whether TLR8-mediated activation mimics bacterial stimulation in the intra-hepatic environment, we pulsed LDCs with ssRNA40, riboflavin-synthesizing bacteria (*P. aeruginosa*), anti-CD3/CD28-coupled beads or PMA/Ionomycin and characterized cytokines produced by hepatic MAIT cells after overnight incubation. Fig. 5 shows that ssRNA40 elicited a cytokine profile in intrahepatic MAIT cells similar to that induced upon bacterial infection, but different to those obtained by anti-CD3/CD28-coupled beads or PMA/Ionomycin. MAIT cells exclusively produced IFN-γ without a concomitant major production of TNF-α (Fig. 5) or IL-17A (not shown) when stimulated with ssRNA40 or bacteria. In contrast, significant proportions of TNF-α mono-producers and IFN-γ/TNF-α double producers were observed only when MAIT cells were stimulated with anti-CD3/CD28-coupled beads or PMA/Ionomycin. Taken together, ssRNA40 induced an intrahepatic immune response that resembles the one triggered by bacterial infection.

### TLR8-activated immune response in diseased livers

The demonstration that a TLR8 agonist, through production of IL-12 and IL-18 from intrahepatic monocytes, stimulated a robust production of IFN-γ in liver resident lymphocytes could have therapeutic implications. In HBV transgenic mice, for example, IL-12 and IL-18 treatment causes an inhibition of HBV replication that was mediated by IFN-γ production from NK and NKT cells [15], [16]. However, pathological processes can alter the cellular composition and the cytokine milieu of the liver microenvironment which can result in the inhibition of T- and NK-cell function [17]. A recent characterization of the immunological profile in chimpanzees treated with an oral TLR7 agonist has for example shown a reduced cytokine and chemokine production in chronic HBV-infected animals in comparison to the healthy ones [18]. We therefore tested whether liver pathological processes can alter the ability of TLR8 agonist to activate intrahepatic immunity.

**Figure 6:**
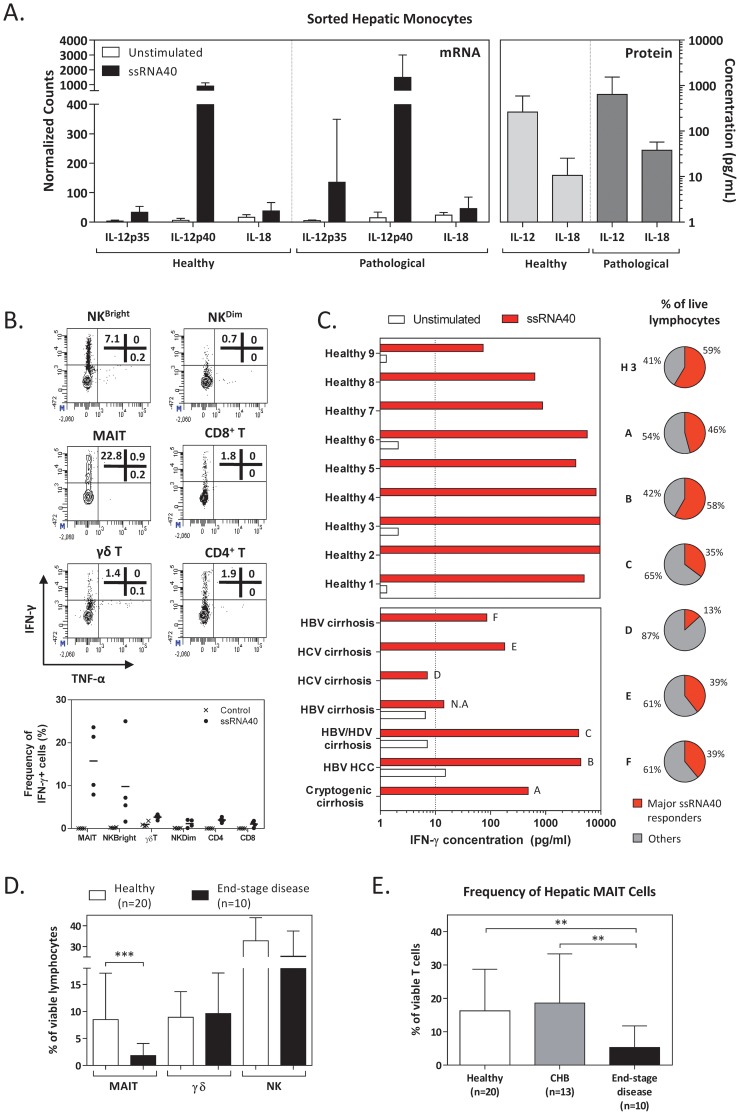
Characterisation of TLR8-mediated immune responses in pathologic intrahepatic immune cells. *(A)* The left panel shows the expression of *IL-12p35*, *IL-12p40* and *IL-18* mRNAs in both healthy (n = 2) and pathologic (n = 5) intrahepatic monocytes before and after stimulation with ssRNA40. Data expressed as mean normalized counts±SD. The right panel shows the concentration of IL-12 and IL-18 in the supernatant after stimulation of intrahepatic monocytes with ssRNA40 from healthy (n = 2) and pathologic (n = 4) donors. Values expressed as mean±SD after subtracting unstimulated background. *(B)* Contour plots illustrate the production of IFN-γ and TNF-α by six immune cell subsets within the liver compartment upon ssRNA40 stimulation. Data from one representative patient are shown as frequency of cytokine-producing cells within each subset. Bottom panel shows the frequency of IFN-γ+ cells within each subset in four patients with end-stage liver disease before and after stimulation with ssRNA40. Each symbol represents one patient and the means are shown as horizontal bars. *(C)* Bar charts depict the secretion of IFN-γ by liver-derived cells from healthy donors (n = 9) and patients with advanced liver disease (n = 7) before and after stimulation with ssRNA40. The pie charts show the frequency of major ssRNA40-responding cells (MAIT, NK and γδ T cells) among viable lymphocytes in the liver. A representative healthy donor (H3) and pathologic donors (A–F) are shown. N.A. denotes that the frequency of effector cells in the patient with HBV cirrhosis was not determined. (*D*) Mean frequency of MAIT, γδ T and NK cells in healthy (n = 20) and end-stage pathologic liver (n = 10) tissues (*** indicates P<0.001). (*E*) Mean frequency of MAIT cells in the liver compartment of healthy (n = 20), untreated CHB patients (n = 13) and patients with end-stage liver disease (n = 10). Values are given as a proportion of viable T cells.

The limited number of cells obtained in diagnostic liver biopsies is insufficient to perform any functional analysis. Thus we purified hepatic immune cells from HBV- or HCV-associated liver explants due to the end-stage liver disease following the identical cold perfusion method applied for healthy livers. In all cases, the pathological processes were very advanced, i.e., either cirrhosis or hepatocellular carcinoma. The functionality of the sorted whole monocytes from pathological liver explants to respond to ssRNA40 stimulation was first evaluated at protein (4 pathologic livers) and mRNA (5 pathologic livers) levels. Upon ssRNA40 overnight stimulation, pathological liver-derived monocytes were able to produce IL-12 and IL-18 cytokines. The cytokine concentrations in pathological livers were slightly higher than observed in healthy livers, even though they didn't reach statistical significance (Fig. 6A).

We next tested the ability of pathological LDCs to produce IFN-γ after ssRNA40 stimulation. Intracellular analysis of IFN-γ production confirmed that also in responding pathological livers, MAIT and NK^Bright^ cells were the major IFN-γ-producing innate immune cells (Fig. 6B). In most of the LDCs of pathological livers (5 out of 7), ssRNA40 stimulated IFN-γ production from viable intrahepatic lymphocytes even though to levels lower than those observed in healthy LDCs (mean±SD: 1292±1957 vs 4975±3948, P = 0.0311; Fig. 6C). Taken together, these data show that ssRNA40 can mediate activation of intrahepatic immunity also in livers with advanced pathological process.

Since the lower IFN-γ quantity detected in the advanced pathological livers might be a direct consequence of the low intrahepatic frequencies of cells responding to ssRNA40, we characterized the composition of liver-derived cells in samples from both end-stage liver diseases and in less advanced liver pathologies, such as chronic hepatitis B. In the latter, low cell numbers have severely limited our analysis only to the frequencies of MAIT cells.

Among the major ssRNA40-responding cells (MAIT, γδ T and NK cells), patients with end-stage liver disease have a significantly lower frequency of MAIT cells compared to healthy subjects (Fig. 6D). In addition, a viable population of monocytes were clearly detectable in LDCs purified from pathological livers with the only difference being an increased frequency of CD16^+^ monocytes (comprising both CD14^+^CD16^+^ and CD14^dim^CD16^+^ subsets) in pathological compared to healthy livers (15.3% vs 5.5% of total CD45^+^HLA-DR^+^ cells; P = 0.05), confirming previous results obtained in chronic hepatitis [19].

MAIT cells were, similar to NK^Bright^ cells [20], enriched in the liver compartment of untreated CHB patients and, unlike in end-stage liver disease, hepatic MAIT-cell frequency remains similar to that found in healthy subjects (Fig. 6E). Thus, the intrahepatic innate lymphocyte population is relatively conserved at least in chronic HBV-infected livers and might be potentially triggered by ssRNA40 stimulation.

## Discussion

We demonstrate here that the human liver environment is specifically equipped with a network of innate immune cells (monocytes, MAIT and NK^Bright^ cells), which is not intrinsically tolerant or hyporesponsive. On the contrary, this cellular network can sense distinct pathogen-associated molecules and can generate a robust immune response. We show that substantial intrahepatic production of IFN-γ is preferentially activated in LDCs by the TLR8 agonist ssRNA40 and we identified CD161^Bright^ MAIT and NK^Bright^ cells as the principal liver-resident IFN-γ-producing lymphocytes. We also show that their activation is mediated by IL-12 and IL-18 produced by the TLR8-activated hepatic monocytes. The demonstration that intrahepatic immune cells were strongly activated by ssRNA40, but not by exposure to LPS, flagellin or other bacterial products, shows that the immune cells residing within the liver vascular system modulate their immunological responses to different pathogen-associated molecules [3]. In this context the TCR specificity of the MAIT cells is not critical and indeed the CD161^Bright^ CD8^+^ T-cell population as a whole, of which the MAIT cells are the key subset, possesses the innate feature to preferentially respond to cytokine-mediated stimulation [13].

Even though TLR8-mediated recognition has been associated with viral infections, new findings link TLR8 sensing to viable bacterial infection [21], [22] and unmodified RNAs, a hallmark of viable bacteria [23], [24]. We might therefore hypothesize that the strong activation of intrahepatic immunity selectively triggered by TLR8 agonist reflects the ability of intrahepatic cells - in particular MAIT cells well known for their antimicrobial capability [25] - to control active bacterial infection while ignoring intestinal floral products that leak into the intrahepatic blood circulation. The strong activation of intrahepatic immunity observed in our experiments utilizing three different bacteria strains (*P. aeruginosa*, *E. coli* and *E. faecalis*) further support such an hypothesis. Nevertheless, our data might indicate that TLR8-mediated activation of intrahepatic immunity might also take place during other pathological conditions, e.g., acute viral hepatitis and hepatic flares during chronic viral hepatitis. Unmodified RNAs, the possible trigger of TLR8-mediated immune response, are also highly enriched within mitochondria and as such can be released during flares when cells are damaged or die [23]. In this context, our observation that ssRNA40-mediated activation could be detected in pathologic liver-derived cells suggests that the ssRNA40-generated immune response can be induced during chronic viral infection even though the impact of hepatotropic viruses on the TLR8-mediated activation of intrahepatic immunity requires more in depth analysis. At the moment, our attempts to activate hepatic MAIT cells (as well as NK^Bright^) by exposing LDCs to purified HBV virions (unpublished results) have been unsuccessful.

Irrespective of their biological role during infections, the network of innate immune cells able to respond to TLR8 agonist within the liver sinusoids can open new therapeutic opportunities. TLR7 agonists are the most studied TLR agonists for the treatment of liver pathologies [18], [26] due to their ability to suppress HCV replication in hepatocytes [27] and stimulate robust IFN-α production in plasmacytoid dendritic cells [28]. However, our data shows that a TLR8 agonist could be advantageous for liver-specific treatment as such an agonist preferentially activates innate immune cells within the liver compartment while TLR7 agonists do not display any preference and do not induce robust IL-12 and IFN-γ production. The selective liver-localized production of IFN-γ could exert an efficient antiviral effect, as HBV clearance was shown to be mediated by IFN-γ in infected chimpanzees [29] and HBV transgenic mice [30]. In this respect it is important to note that the recent demonstration of therapeutic efficacy in HBV-infected chimpanzees of a novel synthetic TLR agonist (GS-9620; labeled as a TLR7 agonist) was not only associated with serum detection of IFN-α, but also of increased IL-12 and CXCL10 levels [18]. Such a cytokine/chemokine profile is also consistent with a TLR8-mediated response and can be explained by the TLR7/TLR8 degeneracy of this synthetic compound at high doses [31].

In addition to its ability to activate NK^Bright^ and MAIT cells, IL-12 production following TLR8 activation might have other immunomodulatory effects, since IL-12 can induce partial functional recovery of exhausted HBV-specific CD8^+^ T cells [32]. This concept is also supported by data from a murine model of persistent HBV infection where single-stranded RNA could reverse immune tolerance [33].

On the other hand, the robust activation of intrahepatic cellular immunity via TLR8 needs to be tightly controlled, both during natural infection or if a TLR8 agonist is to be exploited for therapy of chronic viral infections. IFN-γ is not only an antiviral cytokine but also further induces production of inflammatory chemokines, e.g., CXCL10, by hepatocytes [34]. CXCL10 can induce rapid recruitment of circulating memory T cells into solid organs [35] but can also aggravate inflammatory events that may lead to severe liver damage [36]. In this respect, the balance between protective or damaging effects of intrahepatic innate immune-cell activation will have to be carefully evaluated *in vivo*.

In conclusion, we demonstrate that a network of hepatic innate immune cells responds to TLR8-mediated activation by inducing a potent immune response. The strategic positioning of such cells in the liver can constitute an advantage to immediately sense and control the presence of viable bacteria in the gastrointestinal circulation and might also open new therapeutic options for the treatment of different liver diseases.

## Materials and Methods

### Human samples

Collection of healthy human LDCs (n = 24) and PBMCs (n = 7) was performed as previously reported [8]. Due to ethical considerations, immune cells derived from healthy liver donors were instead compared to those of age-matched healthy donors of PBMCs [8]. Liver biopsies were performed on untreated chronic HBV-infected/CHB patients (n = 13). These biopsies were cultured in medium for 24 hours to gently release mononuclear cells, (‘walk-out’ method) [37]. Cell viability collected using this method exceeded 80%. Among these CHB patients, 10 subjects donated blood as well, from which PBMCs were collected. Mononuclear cells were also collected from HBV/HCV-related liver explants due to end-stage liver disease through perfusion using cold buffer solution (n = 6).

### Ethics statement

All participants in this study are adults. The study was approved by the local ethical boards of the Asian American Liver Centre and the Royal London Hospital and all participants gave written informed consent.

### Antibodies and reagents

Monoclonal antibodies of anti-human-CD3-eFluor605NC (clone OKT3) or –FITC or PE-Cy7 (UCHT1), anti-CD4-eFluor650NC (RPA-T4), anti-CD7-FITC (4H9), anti-CD161-PerCP-Cy5.5 (HP-3G10), anti-HLA-DR-AlexaFluor700 (LN3) were obtained from eBioscience (San Diego, CA). Anti-CD8-V500 (RPA-T8), anti-CD14-PE-Cy7 (M5E2), anti-CD45-V500 (HI30), anti-CD56-PE-Cy7 (B159), anti-IFN-γ-V450 (B27), anti-TNF-α-APC (6401.1111) antibodies were obtained from Becton Dickinson (BD, San Jose, CA). Anti-CD16-APC-Cy7 (3G8), anti-CD19-FITC (HIB19), anti-CD20-FITC (2H7), anti-CD56-FITC or APC-Cy7 (HCD56), anti-Vα7.2 TCR-PE or -APC (3C10) antibodies were obtained from Biolegend (San Diego, CA). Anti-CD161-APC (191B8) and IFN-γ-FITC (45-15) antibodies were obtained from Miltenyi Biotec (UK). Anti-TCRγδ-FITC or -PE (5A6.E9) antibody and Live/Dead Fixable Dead Cell Stain Kit (yellow) were obtained from Invitrogen (Carlsbad, CA). Agonists for human TLR1/2 (Pam3CSK4; 1 µg/mL), TLR2 (HKLM; 10^8^cells/mL), TLR2/6 (FSL-1; 1 µg/mL), TLR3 (Poly(I:C); 10 µg/mL), TLR4 (*E. coli K12* LPS; 1 µg/mL), TLR5 (*S. typhimurium* flagellin; 1 µg/mL), TLR7 (Imiquimod; 5 µg/mL), TLR8 (ssRNA40; 1 µg/mL or otherwise stated) and TLR9 (CpG ODN2216; 5 µM) were obtained from Invivogen (San Diego, CA). Anti-CD3/CD28-coupled beads were obtained from Invitrogen (1∶1 bead-to-cell ratio). Brefeldin A (2 µg/mL for overnight incubation or otherwise stated), bafilomycin A1 (10 nM), chloroquine diphosphate (5 µg/mL), PMA (2 ng/mL) and Ionomycin (1 µg/mL) were obtained from Sigma-Aldrich (Saint Louis, Missouri).

### Quantification of secreted cytokines

LDCs or PBMCs were plated at 100,000 cells per well in 96-well plates and stimulated with TLR agonists or anti-CD3/CD28-coupled beads for 18 hours. Sorted monocytes were plated at 150,000 cells per well in 96-well plates and stimulated with ssRNA40 for 18 hours. Supernatant was collected subsequently and cytokine concentrations were assessed via cytokine multiplex bead-based assay according to the manufacturer's instructions (Luminex, Austin, TX). ELISA of IL-18 was performed according to the manufacturer's instructions (R&D Systems).

### Flow cytometry

The experiment was performed as previously reported [8]. Data was analysed using FACSDiva (BD), FlowJo (Tree Star) or Kaluza (Beckman Coulter) software.

### Cell sorting and depletion

Hepatic MAIT cells were sorted as previously reported [8]. Subsets of hepatic lineage positive (CD3^+^CD7^+^CD56^+^) and antigen-presenting cells (CD3^−^CD7^−^CD56^−^HLA-DR^+^) were sorted using a BD FACS Aria. Subsets of hepatic APCs were subsequently sorted based on the expression of CD14^++^ and CD16^+^ (monocytes), CD20^+^ (B cells), CD11c^+^ (mDCs) or CD123^+^ (pDCs). Pan monocytes were depleted from the bulk population using the pan monocyte isolation kit (Miltenyi Biotec).

### Direct co-culture assay

Sorted hepatic lineage positive cells were co-cultured with sorted APCs at an effector-to-target (E∶T) ratio of 1∶4 in the presence of ssRNA40 overnight with addition of brefeldin A (10 µg/mL) in the final 5 hours. The intracellular production of IFN-γ was assessed by staining and FACS as above.

### Blockade of MR1 or of IL-12 and IL-18 upon ssRNA40 stimulation

LDCs or PBMCs were stimulated with ssRNA40 overnight in the presence of anti-MR1 monoclonal antibody at 10 µg/mL (clone 26.5, kindly provided by Ted. H. Hansen). Alternatively, the stimulated cells were cultured in the presence of anti-IL-12p70 monoclonal antibody (clone 24910) or rhIL-18 binding protein (clone 136007) at concentration 20 µg/mL (R&D Systems). Brefeldin A (10 µg/mL) was added in the final 5 hours. The intracellular production of IFN-γ was determined by flow cytometry.

### Bacterial stimulation of MAIT cells

The bulk population of liver-derived cells was stimulated overnight with two groups of bacteria, i.e., the ones that synthesize riboflavin (*Escherichia coli* or *Pseudomonas aeruginosa*) and the one that does not (*Enterococcus faecalis*). Two million paraformaldehyde-fixed *E. coli* or *E. faecalis* were added for 20 hours in the presence or absence of blocking antibodies against IL-12p70 (R&D Systems) and IL-18 (MBL International, USA) at concentration 5 µg/mL or against MR1 (10 µg/mL) as indicated. Alternatively, to characterize the early and late activation of MAIT cells, THP1 cell line was used as APCs. To assess the cytokine produced by MAIT cells upon infection with riboflavin-synthesizing bacteria, viable *P. aeruginosa* was added overnight at MOI 10 per monocyte. Brefeldin A (10 µg/mL) was added for the final 4–5 hours of culture. Intracellular cytokine staining was performed to detect production of IFN-γ and TNF-α by MAIT cells.

### Statistical analysis

The nonparametric Mann-Whitney *U* test was used to determine the statistical significance of differences.

## Supporting Information

Figure S1 IFN-γ and TNF-α production by MAIT cells upon ssRNA40 stimulation in the presence or absence of chloroquine or bafilomycin A1.
(TIF)

Figure S2 Frequency of IFN-γ-producing cells within MAIT cells upon activation with ssRNA40/Lyovec, ssRNA41/Lyovec, or Lyovec alone at 1 µg/mL (n = 3; mean±SEM; * indicates P<0.05).
(TIF)

Figure S3 Early and late activation of liver-derived MAIT cells by riboflavin-synthesizing and non-riboflavin-synthesizing bacteria. THP1 cells were incubated overnight with *E. coli* or *E. faecalis*. Liver-derived cells were either added for the final 5 hours of incubation (*A and B*) or were co-cultured overnight (*C and D*). The frequency of IFN-γ-producing MAIT cells (*A and C*) and the ratio of the frequency of IFN-γ-producing MAIT cells in the presence of blocking antibody to that in the absence of any blocking antibody are displayed (*B and D*; n = 3; each symbol represents one donor). The frequency of IFN-γ-producing MAIT cells upon bacterial infection without any blocking antibodies was set as 1.
(TIF)
